# Aligning with regulatory agencies for the use of digital health technologies in drug development: a case study from Parkinson's disease

**DOI:** 10.3389/fdgth.2025.1415202

**Published:** 2025-10-23

**Authors:** Derek L. Hill, Camile Carroll, Ramona Belfiore-Oshan, Diane Stephenson

**Affiliations:** ^1^ Biomedical Engineering, University College London, London, United Kingdom; ^2^ Translational and Clinical Research Institute, Newcastle University, Newcastle, United Kingdom; ^3^ National Institute for Health and Care Research (NIHR) Newcastle Biomedical Research Centre (BRC), Newcastle University, Newcastle, United Kingdom; ^4^ Critical Path Institute, Tucson, AZ, United States

**Keywords:** drug development, digital health technologies, data sharing, regulatory framework, Parkinson's disease, neurological disorders

## Abstract

Digital Health Technologies (DHTs) have been under investigation for many years as innovative tools for Parkinson's disease motor symptoms given their inherent high-frequency, sensitive, and objective measurement properties. DHTs used in drug development, can be defined as Drug Development Tools (DDT), though some DHTs may also be categorized as medical devices. The recent rapid increase in use of DHTs in clinical trials has been accompanied by a rapidly evolving regulatory landscape, resulting in a challenging environment for widespread implementation of DHTs in applications that will provide clear impact on pharmaceutical company drug development pipelines. Parkinson's disease represents a disease of escalating burden with high unmet need for therapies that are disease modifying. Early intervention is a key area of focus, yet the heterogeneity of symptoms and lack of biomarkers poses challenges for drug development. Furthermore, the technologies and device platforms, both hardware and software, are rapidly evolving, and the companies developing the underlying devices frequently have objectives and timelines that may not align with those of the pharmaceutical industry. DHTs therefore have a unique set of challenges in terms of devising meaningful measures, standardization of data collected, responding to evolving regulatory expectations, and ensuring alignment across stakeholders. There is a growing need for new models of collaboration to bring together diverse stakeholders required to achieve regulatory endorsement of DHTs for use as DDTs. Collaborations between stakeholders working on DHTs need to be firmly anchored in the regulatory ecosystem as many regulatory challenges in DHTs have parallels in other technologies. Furthermore, there is an especially urgent need to define the pre-competitive space in which DHT data can be shared, data collection standards devised, and novel analysis approaches that are robust to residual variability developed. Critical Path for Parkinson's Consortium's (CPP) Digital Drug Development Tool (3DT) initiative is highlighted as a case example to illustrate how pre-competitive public private partnerships can advance the regulatory maturity of digital health technology measures for use in clinical trials.

## 1 Introduction

Digital Health Technologies (DHTs) used as Drug Development tools (DDTs) represent an important example of a regulated technology to support medical product development. These technologies have the potential to meet pharmaceutical industry needs for high frequency, sensitive, and objective measures of a patient's disease progression, and a patient's response to treatment in real-world settings (1).

DHTs have attracted particular interest in chronic progressive diseases of the nervous system (2). This is due to the heterogenous nature of symptoms, slow insidious onset of symptoms with long duration of presymptomatic underlying disease, and lack of patient centered measures that can be used to define true impact of novel therapies on patient's quality of life.

DHT measures may therefore accelerate the development of new drug and biological therapies in areas of unmet medical need and enable these treatments to be better focused on treating the aspect(s) of disease of most importance to patients.

DHTs, when used to support drug development, sit at the interface between medicine and device regulations. The applicable regulatory landscape is rapidly evolving including across regulatory authorities. Here we make use of terminology from the FDA's recent guidance document on Digital Health Technologies for Remote Data Acquisition (3), and limit our discussion to DHTs that incorporate sensors (e.g., motion sensors). We use the term “DHT measure” to mean the output of a DHT used as a drug development tool, and “DHT Device” for the data collection device e.g., wearable sensor from which the DHT measure is obtained or derived.

In this paper, we describe the work of Critical Path Institute's (C-Path) Critical Path for Parkinson's (CPP) Digital Drug Development Tools (3DT) initiative to collect evidence that DHTs can reliably and accurately measure PD progression at early stages in drug naïve patients over one year duration, in order to advance the regulatory maturity of DHTs for assessing patients with Parkinson's disease (PD). CPP is a public private partnership focused on the development of drug development tools targeting early stages of the disease. The key milestones for DHTs being used as DDTs are (a) letter of support and (b) qualification. A letter of support is issued by the medicines regulator to describes the regulator's thoughts on the potential value of a DDT and encourages further evaluation. A DDT qualification is a public regulatory opinion that encourages the use of a qualified DDT for a specific context of use to expedite drug development and review of regulatory applications.

The regulatory landscape for DHTs has been evolving significantly since CPP was launched in 2015 (4): there has been a rapid increase in the response of regulators to the needs of DHTs and their use in drug development. Regulatory agencies have published several guidance and discussion documents focused on DHTs and with some DHT measures reaching a high level of maturity with certain regulators. This regulatory framework enables DHTs to be used on a protocol-specific basis, or to be qualified for more general application in a context of use. Many DHT measures are generated using machine learning (ML) and artificial intelligence (AI), which means they may be impacted by AI-specific regulations being proposed in several jurisdictions, including the European Union AI Act (5).

Table 1 shows the timeline for advances in the regulatory landscape over the past several years both in U.S. and Europe, with key regulatory publications from the U.S. Food and Drug Administration (FDA) and European Medicines Agency (EMA) highlighted. Although primarily focused on regulation of medicinal products, we include cybersecurity guidance documents focused on medical devices generally relevant to all DHTs, whether medical devices or not. Notably, the FDA's March 2024 “AI & Medical Products” guidance specifically describes how medicines and device regulators are working together in this rapidly evolving area.

**Table 1:** Recent key regulatory guidance and frameworks relevant to DHTs. The majority are published by medicines regulators though cybersecurity guidance documents published by medical device regulators are also included.

Date	Regulator	Title	Comment	Link
June 2018	FDA	Patient-Focused Drug Development: Collecting Comprehensive and Representative Input	Guidance document	https://www.fda.gov/regulatory-information/search-fda-guidance-documents/patient-focused-drug-development-collecting-comprehensive-and-representative-input
June 2020	EMA – Human Medicines Division	Questions and Answers: Qualification of Digital Technology-Based Methodologies to Support Approval of Medicinal Products	Document to support qualification of DHT methodologies	https://www.ema.europa.eu/en/documents/other/questions-and-answers-qualification-digital-technology-based-methodologies-support-approval-medicinal-products_en.pdf
July 2020	EMA – Medical Device Coordination Group	MDCG 2019–16 Rev.1 Guidance on Cybersecurity for medical devices	Guidance Document	https://health.ec.europa.eu/document/download/b23b362f-8a56-434c-922a-5b3ca4d0a7a1_en
February 2022	FDA	Patient-Focused Drug Development: Methods to Identifying What Is Important to Patients	Guidance document	https://www.fda.gov/regulatory-information/search-fda-guidance-documents/patient-focused-drug-development-methods-identify-what-important-patients
June 2022	FDA	Patient-Focused Drug Development: Selecting, Developing, or Modifying Fit-for-Purpose Clinical Outcome Assessments	Guidance document	https://www.fda.gov/regulatory-information/search-fda-guidance-documents/patient-focused-drug-development-selecting-developing-or-modifying-fit-purpose-clinical-outcome
April 2023	FDA	Patient-Focused Drug Development: Incorporating Clinical Outcome Assessments Into	Guidance document	https://www.fda.gov/regulatory-information/search-fda-guidance-documents/patient-focused-drug-development-incorporating-clinical-outcome-assessments-endpoints-regulatory
May 2023	FDA - CDER	Artificial Intelligence for Drug Development	Informational	https://www.fda.gov/about-fda/center-drug-evaluation-and-research-cder/artificial-intelligence-drug-development
May 2023	FDA - CDER	Using Artificial Intelligence & Machine Learning in the Development of Drug & Biological Products	Discussion Paper/Request for Feedback	https://www.fda.gov/media/167973/download
March 2023	FDA	Framework for the Use of Digital Health Technologies in Drug and Biological Product Development	Framework; PDUFA VII	https://www.fda.gov/media/166396/download?attachment
March 2023	EMA – GCP IWG	Guideline on computerised systems and electronic data in clinical trials	Guidance Document	https://www.ema.europa.eu/en/documents/regulatory-procedural-guideline/guideline-computerised-systems-and-electronic-data-clinical-trials_en.pdf
Sept 2023	FDA	Cybersecurity in Medical Devices: Quality System Considerations and Content of Premarket Submissions	Guidance Document	https://www.fda.gov/regulatory-information/search-fda-guidance-documents/cybersecurity-medical-devices-quality-system-considerations-and-content-premarket-submissions
Dec. 2023	FDA	Digital Health Technologies for Remote Data Acquisition in Clinical Investigations	Guidance Document	https://www.fda.gov/regulatory-information/search-fda-guidance-documents/digital-health-technologies-remote-data-acquisition-clinical-investigations
Jan 2025	FDA	Considerations for the Use of Artificial Intelligence To Support Regulatory Decision-Making for Drug and Biological Products	Draft Guidance	https://www.fda.gov/media/184830/download
June 2025	FDA	Cybersecurity in Medical Devices: Quality System Considerations and Content of Premarket Submissions	Guidance document	https://www.fda.gov/media/119933/download

## 2 Unique challenges of DHTs

Regulators have made much progress in provision of guidance for DHTs in drug development, though the impact of DHTs in clinical trials has so far been limited; for example, no drug has yet been approved by the FDA based on a DHT derived primary endpoint (6) and the EMA has recently described regulatory experience with DHTs in the context of registrational studies as minimal (7). Issues relate to the rapid rate of innovation in digital technologies, the types of companies in the ecosystem, and the intersection between regulations related to clinical trials, medical devices, and data protection/privacy.

### 2.1 Rapid rate of innovation

The rapid rate of innovation in the technologies incorporated in DHTs (e.g., sensors, ML algorithms, connected devices) means that the product lifecycle of a DHT is often a small number of years. A DHT may rely on consumer computing platforms such as smartphones. The lifetime of DHT devices, and sometimes even digital companies, is short compared to the timescale of drug development. It is therefore hard for DHTs to “travel with a molecule” from phase I to approval, which might be a period of more than 10 years. Even if a particular hardware remains stable, the installed software might periodically upgrade in ways that make the data non-comparable.

### 2.2 Standardization and harmonization

A consequence of the rapid rate of innovation in the hardware, software and measurements from DHTs is the need to obtain comparable data across time and studies. The diversity in technologies available, the speed of innovation, including software upgrades and new versions of hardware, and the proprietary nature of some algorithms means that obtaining comparable data is a considerable challenge.

One state-of-the-art approach in this area has been described by the Mobilise-D consortium (8), in which multiple types of motion sensors have been compared against a gold standard in a laboratory setting. This highlighted considerable remaining challenges in standardizing DHT data even from accelerometers, which are arguably the most mature of DHT sensor technology. The authors suggest guidelines to assist standardization efforts for future studies.

Parallels have previously been drawn between DHTs and imaging. Putting in place suitable standardization has been important in the development of neuroimaging in clinical trials (9, 10) and is a focus of the FDA guidance on imaging endpoints in clinical trials (11). It is important to note that, while there are parallels with imaging, DHTs are used for remote data acquisition (e.g., in the home) and there is considerable additional variability compared to that of the in-clinic controlled environment applicable to imaging. This puts additional requirements on the standardization of DHTs that allow for bridging in-clinic with at-home measurements. Standardization of a particular DHT measure, therefore, should consider implications of hardware, software, and measurement environment. The experience of standardizing imaging endpoints encourages the standardization to be done in the context of a specific measurement such as hippocampal volume (12) or Positron Emission Tomography standardized uptake value (PET SUV) (10), and for measurements obtained from diverse scanners (sometimes with contrast or tracers) and algorithms to be compared in terms of effect size in a relevant comparison e.g., separating diseased from normal or progressing from non-progressing subjects (13, 14). Once a measurement is clearly defined, the standardization task is easier to specify. The lack of consensus on specific DHT measures has been a barrier to progress in this measurement-driven standardization. Because some DHT devices can generate multiple possible DHT-derived measures (for example the output from a wrist-worn accelerometer could be used to calculate measures of gait, tremor or sleep), the appropriate standardization and algorithm validation should be measure rather than device specific.

### 2.3 Business models and data protection and privacy

Technology companies, whether focused on digital health or consumer tech, frequently have business models that involve monetizing data (15). Sophisticated consumer hardware and software used by individuals is provided at low cost (and for software, often free) in exchange for the user agreeing to transfer their data to the tech company and give ownership, or at least wide-ranging rights to use it for commercial purposes. The huge volumes of data thus acquired by the tech companies can be used to improve the product, but also can be sold freely, so an individual's data may be used by unknown third parties for purposes that were neither pre-defined nor specifically consented to by the user. These data-centric business models are potentially incompatible with the desire of pharmaceutical companies, healthcare providers, and regulators to ensure that patient data is carefully controlled and only used for pre-specified purposes with informed consent.

### 2.4 Intersection between different regulatory systems

A further challenge relates to DHTs operating at the interface between different regulatory frameworks. Many DHT devices (e.g., smartphones and smartwatches with fitness apps and activity trackers) are designed for consumer use and have limited regulatory oversight. A sub-set of DHT devices are either medical devices or contain software components that are “software as a medical device”. Use of any of these DHT devices in clinical trials adds new regulatory requirements around validation of computer systems that come from Good Clinical Practice (GCP) (16) (21CFR11 in USA, Annex 11 of the Clinical Trial Regulation in Europe). The EMA has made clear in recent publications that GCP regulations around validation and audit trail apply to mass market wearables and mobile phones (17). Some digital health companies struggle to put in place systems that are compliant with these requirements and do not see a business case for achieving compliance, given the small size of the clinical trial market for most of these companies.

The need for different models of data use, and the requirements of validation and audit trail, mean that commercial collaborations between the pharmaceutical and tech sectors can be challenging.

This further emphasizes that for DHTs to have a significant impact on the development of new treatments, new models of collaboration are needed. There is also a need to acknowledge that the price point of the technologies used in clinical trials is likely to be significantly higher than the prices that end-users are used to for consumer digital technologies.

## 3 The need for new models of collaboration to develop DHTs

In recent years, there has been significant optimism that “digital” technologies could rapidly impact drug development, and as a result, relevant industry and public organizations are investing in DHTs across various therapeutic areas. There has been an associated rapid increase in the number of clinical studies incorporating DHTs (2), particularly in chronic progressive disorders of the nervous system where the failure rate is high and there is a lack of sensitive, clinically meaningful DDTs. The application of DHTs to disorders of the nervous system is growing at a rapid rate with Parkinson's being most prominent of all (Evidence from https://www.ClinicalTrials.gov on the growth of Digital Health Technologies in neurology trials (2).

It is increasingly clear that while DHTs have great potential to positively impact drug development, the timescale of their development has not proved to be rapid in comparison to other technologies such as imaging, and at the date of writing, we have not yet seen any new drugs approved based on a DHT measurement. One DHT measure that has achieved the regulatory milestone of being qualified as a primary endpoint in Duchenne muscular dystrophy (DMD) by the EMA is the Stride Velocity 95th centile (SV95C) (18). This effort took more than a decade (19) to complete, which is not indicative of the minimum (or maximum) time required but illustrates the challenges of navigating the regulatory environment for DHTs. While most recent DMD studies have included SV95C as a secondary outcome (NCT05524883, NCT05096221, NCT06138639, NCT05982119, NCT04906460), the use of this measure has been explored for other neuromuscular diseases including Spinal Muscular Atrophy, Facioscapulohumeral muscular dystrophy, and Limb Girdle muscular dystrophy. However, it is still unclear how the learnings from the DMD qualification will be applied or whether they are fully translatable to those other diseases (20).

Many pharmaceutical companies and research institutions have been independently working on developing DHT measures, which has resulted in an explosion of proposed approaches to measuring concepts of interest such as gait (21). It is becoming increasingly clear that the challenges are too big to overcome as individual companies and organizations alone, necessitating a collaborative and harmonized approach. Increasingly, pharmaceutical companies are looking for a clear impact on their drug development programs and adapting their investment in DHTs accordingly. A consortium-based approach is therefore desirable and aligns with regulatory agency recommendations for public-private partnerships to increase their efficiency in advancing DHTs (22, 23). Some industry-led consortia have sought to develop high-impact DHT measures that are disease-agnostic or are cross-disease digital endpoints in areas such as fatigue, sleep (24), and mobility (21). Regulators, however, have consistently communicated that, just as for other (non-digital) technologies, data should be submitted for a single disease and context of use (COU).

It is therefore increasingly important that, for reasons of cost effectiveness and rate of progress, development of DHTs is undertaken collaboratively rather than in isolation, and anchored within organizations that have wide-ranging experience in development of non-DHT DDTs. Some of the DHT challenges identified above could be addressed by means of collaborative data analysis platforms such as federated learning.

## 4 The evolving DHT regulatory landscape

While DHTs have been used in clinical research for decades (25), there has been significant increase in use over the last 5 years particularly post-COVID-19 pandemic, and a rapid evolution in the regulatory landscape for DHTs as DDTs. In particular, there are recent regulatory publications specific to DHTs (3, 26) and those that can apply to DHTs including those on patient-focused drug development, use of AI in devices (27), drug development (28), and validation of computer systems (17).

Industry has proposed the use of DHTs for several applications in drug development that span a variety of different intended uses to enhance decision making in clinical trials, not only as digital endpoints (29). DHTs have potential to be used for advancing novel candidate therapies at all stages of drug development including patient subgroup characterization, optimizing trial design, patient identification and recruitment, risk assessment and adverse event prevention, remote interventions to enable decentralized clinical trials, externally controlled trials, and label indication expansion.

Up until 5 years ago, it was common to refer to all DHT measures as “digital biomarkers”. However, the DHT measures can be used for multiple purposes to support drug development, and as such, the use of DHTs might meet either the definition of a biomarker or of a clinical outcome assessment (30):

• **Digital Biomarker**: “a characteristic or set of characteristics, collected from digital health technologies, that is measured as an indicator of normal biological processes, pathogenic processes, or responses to an exposure or intervention, including therapeutic interventions.” (31)
• **Clinical outcome assessment (COA)**: an assessment of how someone feels, functions, or survives (32).

For some DHT measures, this distinction remains a matter of debate (33). For example, it is possible to argue that change in a measure of gait due to progression or treatment of PD is both “an indicator of a pathogenic process or biological response” and that it is an “assessment of how someone feels or functions”. This distinction has practical applications. For a “biomarker”, the sensitivity of the measure to the pathogenic process or biological response is the priority, with the goal of achieving a larger effect size and hence needing fewer participants and/or less time for a clinical trial for a new medicine, in which demonstration of drug efficacy is the objective. For COA, however, clinical meaningfulness is the priority, and a sensitive measure that is not meaningful to the participant or their physician would be considered inappropriate in a trial in which the objective is demonstration of clinical effectiveness. This has implications for the types of data needed to advance the regulatory maturity of DHTs. The next section discusses the regulatory focus on patient-focused drug development, which is of great relevance to the use of DHTs for COAs.

### 4.1 Patient-focused drug development

Medicines regulators have an increasing focus on ensuring that data collected during clinical trials of new medicines takes account of the patients' voice. The FDA's recent series of guidance documents on patient-focused drug development (34–37) refer to DHTs in various places, and it is clear that regulators will treat many DHT measures as a type of Clinical Outcome Assessment (COA). The implication for the use of DHTs in clinical trials is that regulators want to see evidence that the DHT measure is relevant to a meaningful aspect of health for the patient. For example, accelerometers have become ubiquitous for tracking activity in smartphones and smartwatches. There are established ways of calculating “activity metrics” from this acceleration data, e.g., step count, cadence and amount of vigorous activity, and many novel motion-sensor-derived measures can be developed using machine learning and artificial intelligence. The focus on meaningfulness of DHT measures means that it is necessary to show that the DHT measure can be linked to a concept of interest relevant to the condition, and a meaningful aspect of patient health. This approach is being followed by consortia working in some disease areas e.g., nocturnal scratch (38). This linkage between DHT measure and meaningful aspects of health needs to be shown for each clinical condition, and regulatory agencies have similar expectations as to data required for drug development tools such as biomarkers and COAs (e.g., both observational and clinical trial data to support a defined COU).

Regulators are using the term “fit for purpose” to describe when a DHT measure is ready for use in a clinical investigation, and they make clear that a DHT measure has to be validated for a single COU,: it is considered fit-for-purpose when “the level of validation associated with a medical product development tool is sufficient to support its context of use” (30).

Whether a DHT is fit for purpose is determined by the strength of the evidence in support of interpreting the DHT measure as reflecting the concept of interest within the COU. Fit-for-purpose in the regulatory context means the same thing as valid within modern validity theory, e.g., validity is “the degree to which evidence and theory support the interpretations of test scores for proposed uses of tests” (39).

### 4.2 FDA digital health technology guidance/framework

In 2021, the FDA published a draft guidance, “Digital Health Technologies for Remote Data Acquisition in Clinical Investigations” (40), and subsequently published a framework document that seeks to explain how DHTs fit into FDA's thinking (26); a final version of the DHT guidance was published in December 2023 (3). Key implications of this guidance are that the initial step in choosing an appropriate DHT is to “consider the clinical event or characteristic of the disease or condition of interest that is to be measured, identify appropriate technical and performance specifications of a DHT, and consider the proposed trial population”. In practice, very often innovation in DHTs has started with available DHT devices (e.g., wrist-worn accelerometers) and sought to derive from this DHT device a DHT measure that meets a drug development need. This guidance further emphasizes the need to clearly define a rationale for the selection of a particular DHT for a context of use, the need for appropriate verification, validation, usability assessment, and the consideration of risks, including confounds (they give the example of false positive detection of tremor in PD from a person traveling in a car on a bumpy road). In the framework published, the FDA acknowledged that it needs to adapt internally to be able to properly consider DHTs and provide sponsors with consistent feedback between review divisions.

### 4.3 Machine learning and AI in drug development

Many DHT measures are calculated using machine learning (ML) or artificial intelligence (AI). Developers and users of DHTs therefore need to take account of the evolving regulatory landscape for AI. This is an area of rapid evolution in regulatory thinking and a potentially significant divergence between jurisdictions. The FDA has recently published a discussion paper “Using Artificial Intelligence and Machine Learning in the Development of Drug and Biological Products” (28), which is relevant to DHTs. Of particular relevance is the need to manage risk that arises from use of ML/AI models, which the regulators argue can be distinct from risk in traditional rules-based software. These risks include data quality risks, bias risks (e.g., selection bias, confounding variables), and data security and privacy risks (41).

### 4.4 Recent DHT regulatory milestones

As of August 2025 there are a total of two letters of support and two full qualification opinions from the EMA on the use of DHTs as drug development tools as digital endpoints. The FDA manages a public website (41) showing it has accepted multiple digital endpoints into the COA qualification program for a range of conditions including DMD, Multiple Sclerosis (MS), chronic heart failure, sarcopenia and atopic dermatitis. By reviewing the Agency feedback provided in each case example there are common issues to be addressed even though the specific indication may be different (42). Sharing of such knowledge and learnings promises to catalyze progress and avoid redundancies and inefficiencies.

## 5 Critical path institute's 3DT initiative

C-Path is a not-for-profit organization that has nearly two decades of experience leading public-private partnerships spanning multiple diseases to advance regulatory maturity of drug development tools (Table 2) across several neurological disorders including Alzheimer's disease (AD), PD, and DMD. C-Path-led consortia have achieved regulatory milestones from full qualification opinions to Letters of Support and Fit for Purpose FDA and EMA endorsements (43).

**Table 2:** Critical path institute (C-path) regulatory milestones to date.

Regulator	Letters of support		Qualifications	
	Total	% led by C-Path	Total	% led by C-Path
FDA	25	44%	16	50%
EMA	49	20%	30	30%

FDA, US Food and Drug Administration; EMA, European Medicines Agency, as of March 2024.

The 3DT initiative in Parkinson's disease was launched in 2018 under the auspices of the established global consortium, CPP, as a data-driven collaborative path to share knowledge and resources. The vision of 3DT is to advance the regulatory maturity of DHTs as drug development tools for decision-making in PD trials targeting early Parkinson's disease.

CPP's 3DT initiative has provided a data-driven framework for multiple sponsors who have agreed to collaborate on optimizing the use of DHTs in PD drug development. The 3DT consortium involves sharing of patient-level digital device data (including raw data) with members. The 3DT consortium has maintained regular interaction with medicines regulators, including a Critical Path Innovation Meeting (CPIM) held with the FDA and an Innovation Task Force (ITF) meeting with EMA, both in 2019. Regular additional interactions include with FDA staff members regularly attending monthly consortium meetings, thereby providing an ongoing regulatory dialogue. These interactions with regulators have highlighted several challenges facing the field, including the need for strategies for establishing meaningful clinical endpoints, controlling sources of variability, and evaluating DHT performance in normative as well as diseased cohorts.

A key focus of CPP 3DT is the observational study WATCH-PD (Wearable Assessment in the Clinic and at Home in PD) (NCT03681015) which is focused on an early *de novo* PD target population. This study evaluates the ability of research-grade wearable sensors, a smartwatch and a smartphone to assess key features of PD, using a platform that maps directly onto the MDS-UPDRS. WATCH-PD aims to determine the specific disease features these digital tools can detect, whether the measures differed between individuals with early PD and age-matched controls, and how well the digital measures correlated with traditional ones (44, 45). The CPIM and ITF meetings in 2019 provided regulatory feedback that was used to refine the Watch-PD protocol, adding a normal control arm, and including more rigorous qualitative evaluation of the meaningfulness of the DHT measures to study participants, illustrating the value of early interaction with regulators. CPP recognizes that WATCH-PD is a single study that is noninterventional and has limitations.

### 5.1 3DT progress to date

3DT has brought together a group of leading industry partners, academic key opinion leaders, patient advocacy organizations, and people living with PD from around the world.

The key components and milestones in the phases of 3DT are shown in Table 3.

**Table 3:** Key components of C-path's CPP 3DT.

Regulatory alignment	Data strategy	Patient focused approach	Legal framework
Formal engagement with FDA (CPIM) and EMA (ITF and qualification advice)	C-Path platform for curation and sharing of DHT data, including raw sensor data, within consortia	Included PD-affected individual in WATCH-PD study design	Informed consent for WATCH-PD included data sharing with C-Path
Informal engagement with FDA and EMA regulators at consortium meetings and workshops including joint with EFPIR	Sharing of unprocessed in-clinic and at-home WATCH-PD data while study on-going.	Shared patient-centric trial recommendations using DHT	Data sharing agreements in place with consortium members and C-Path advisors.
Role of C-Path consortia highlighted at 4 workshops hosted by regulators	Sharing of DHT data from pharma sponsored studies.	Data from qualification study shared with patients.	HIPAA and GDPR compliance
Feedback from regulators impacted Watch-PD protocol and analysis plans including addition of control arm	Anonymised Data available to individual sponsors for research and development use only (not commercialization)		
Co-authored abstracts and manuscripts	Analysis design takes account of regulatory feedback.		

EMA, European Medicines Agency; FDA, Food and Drug Administration; EFPIA, European Federation of Pharmaceutical Industries and Associations; ITF, Innovative Task Force; CPIM, critical path innovation meeting; HIPAA, health insurance portability and accountability Act; GDPR, general data protection regulation.

## 6 Discussions and conclusions

There is an evolving regulatory landscape for Digital Health Technologies as drug development tools, with multiple stakeholders independently approaching regulatory agencies for endorsement. Experience of many parallel initiatives approaching regulatory agencies to date suggests that navigating the regulatory path to enable DHTs to have a significant impact on drug development and defining success in addressing drug development needs remain challenging. The experiences of the 3DT consortium highlight the value of collaborative approaches involving pharma industry and academic experts, leveraging Critical Path Institute's experience of advancing the regulatory maturity of a diverse range of drug development tools, from Patient Reported Outcomes (PROs) to imaging biomarkers (46). Tackling challenges collectively by advancing data-driven solutions and sharing costs and risks, as well as embracing open science, can avoid duplication of effort and therefore improve the efficiency with which we advance the regulatory acceptance of DHTs and their use in clinical trials. While DHTs make use of different technologies from those used in other DDTs, C-Path's experience in other types of DDTs, and its existing infrastructure for legal, data, and regulatory engagement has proved valuable in enabling the 3DT consortium to progress. Specific regulatory feedback on the Watch-PD case study itself (such as the need to incorporate a control group, and to add a qualitative element to the study to assess the symptoms of most importance to patients) has informed multiple sponsors as to which considerations are essential across device platforms, both in other PD applications and in different disease areas.

The experiences to date make clear that, while digital technologies have many distinct characteristics, the use of DHT measures as drug development tools needs to fit into the same framework as other DDT technologies. It is therefore essential to precisely define:

• The concept of interest (COI): a clinical event or characteristic of the disease or condition of interest that is to be measured, as either a COA or biomarker.
• The application of the DHT In terms of how it will be applied for drug development decision making (COU). The way the DHT measures the COI will impact the drug development process.
• The rationale for the use of a particular DHT measure relevant to that COI including why it meets the required technical and performance specifications.
• How the selected DHT measure is meaningful.
• The evidence that demonstrates the DHT measure is sufficiently well validated for the COU (“fit for purpose”).

For much work to date on DHT- measures as DDTs, it is hard to precisely define all these elements. A diversity of stakeholders is key to success and spans technology experts, clinicians, industry, academic experts, nonprofit organizations, people with lived experience, and regulators themselves. New approaches and new models of collaboration are needed to advance the field as efficiently as possible to be able to attend to the time-sensitive needs of patients. Such collaborative approaches should learn lessons from other types of DDTs (e.g., imaging) to address challenges of standardization and collaborative implementation of analysis methods to enable convergence rather than divergence of proposed DHT measurements. Given the challenges of integrating and harmonizing legacy data collected across distinct device platforms, it is recommended that precompetitive collaborations focus on sharing risks, costs, and prospective study design and collection to optimize DHT studies for the future. We propose nine crucial next steps to advance the field, as shown in Table 4. While these recommendations are based on experience with this Parkison's disease case study, they are more generally applicable for DHTs used as DDTs in this regulatory environment.

**Table 4:** Nine recommended next steps to take the field forward.

#	Recommendation
1	Define a pre-competitive space in which pharmaceutical companies, device companies, academic experts and people with lived experience can collaborate on specific COIs and COUs.
2	Ensure alignment of incentives for all stakeholders, taking account of differing business models and the need to devise tools that can be deployed in settings with low network bandwidth, limited digital literacy, and in low and middle-income countries.
3	Build on this alignment within the pre-competitive space to enable meaningful sharing of DHT data for defined regulatory purposes, taking into account ethical and pragmatic considerations.
4	Establish good practice for demonstrating meaningfulness of DHT-derived measures.
5	Establish good practice for demonstrating equivalence between different hardware/software for a given DHT measure.
6	Devise standardization approaches in data acquisition, how devices are used in studies, data handling, and data analysis for defined DHT measurements for a COI and catalyze the implementation of these in future studies.
7	Develop collaborative data analytics platforms that are able to handle the large data volumes collected and are designed to be robust to residual variation in data collection given the rapidly evolving and heterogenous nature of DHT hardware and embedded software.
8	Provide a clearer roadmap for demonstrating “fit for purpose” DHTs by focusing on some exemplar measures. Align across parallel consortia to advance multiple data sources synergistically.
9	Define pathways to improve usability to reduce patient and site burden, especially in diverse and global clinical trial populations.
